# Optimizing environmental DNA sampling effort for fish inventories in tropical streams and rivers

**DOI:** 10.1038/s41598-019-39399-5

**Published:** 2019-02-28

**Authors:** Isabel Cantera, Kévin Cilleros, Alice Valentini, Axel Cerdan, Tony Dejean, Amaia Iribar, Pierre Taberlet, Régis Vigouroux, Sébastien Brosse

**Affiliations:** 1 Laboratoire Évolution & Diversité Biologique (EDB UMR5174), Université Paul Sabatier - Toulouse 3, CNRS, IRD, UPS, 118 route de Narbonne, 31062 Toulouse Cedex, France; 2 Irstea, UR RECOVER, Equipe FRESHCO, 3275 Route de Cézanne, CS 40061, 13182 Aix en Provence, France; 3 SPYGEN, 17 rue du Lac Saint-André Savoie Technolac - BP 274, Le Bourget-du-Lac, 73375 France; 4 Écologie des Forêts de Guyane (UMR EcoFoG), Campus Agronomique, Kourou, French Guiana; 5 Laboratoire d’Ecologie Alpine (LECA UMR5553), CNRS, Université Grenoble Alpes, 38041 Grenoble, France; 6 HYDRECO, Laboratoire Environnement de Petit Saut, B.P 823, F-97388 Kourou Cedex, French Guiana

## Abstract

Environmental DNA (eDNA) metabarcoding is a promising tool to estimate aquatic biodiversity. It is based on the capture of DNA from a water sample. The sampled water volume, a crucial aspect for efficient species detection, has been empirically variable (ranging from few centiliters to tens of liters). This results in a high variability of sampling effort across studies, making comparisons difficult and raising uncertainties about the completeness of eDNA inventories. Our aim was to determine the sampling effort (filtered water volume) needed to get optimal inventories of fish assemblages in species-rich tropical streams and rivers using eDNA. Ten DNA replicates were collected in six Guianese sites (3 streams and 3 rivers), resulting in sampling efforts ranging from 17 to 340 liters of water. We show that sampling 34 liters of water detected more than 64% of the expected fish fauna and permitted to distinguish the fauna between sites and between ecosystem types (stream *versus* rivers). Above 68 liters, the number of detected species per site increased slightly, with a detection rate higher than 71%. Increasing sampling effort up to 340 liters provided little additional information, testifying that filtering 34 to 68 liters is sufficient to inventory most of the fauna in highly diverse tropical aquatic ecosystems.

## Introduction

In recent years, environmental DNA (eDNA) metabarcoding has been claimed as a promising tool to estimate biodiversity and its change through time^1,2^. In particular, this technique is employed to identify the free DNA released by organisms in their environment^3^. In aquatic ecosystems, the use of eDNA has been widely developed during the last years and has turned from the detection of specific species of amphibians, fish, mammals, insects and crustaceans^4^ to the detection of whole communities^5–10^. The latter studies besides reconstructing entire aquatic communities of fishes and amphibians, compared the detection performance between eDNA metabarcoding and capture-based sampling methods used to collect specimens in streams and rivers. Through this, they showed that both methods provided similar or more complete species inventories, hence opening avenues to use this method for ecological and conservation studies.

Obtaining biodiversity inventories with eDNA metabarcoding requires several subsequent steps including: DNA sampling and collection, laboratory protocols (DNA purification, marker targeting and sequencing), bioinformatics analyses and taxonomic assignment of sequences. The growing interest in this method resulted in the development of a considerable variety of protocols for each step of the eDNA procedure^11^. This makes comparisons between studies challenging considering that it has been illustrated that the choice of markers^5,8^, DNA collection methods^12,13^ and laboratory protocols^5,12,13^ may influence the detection of aquatic species. Furthermore, the environmental conditions and the targeted taxon can also affect detection rate because eDNA release varies among taxa^12,14^ and water physiochemical factors may impact eDNA degradation^15,16^. Therefore, the performance of biodiversity detection in the water depends on a combination of protocols choice, as well as the environmental conditions and the targeted taxonomic group.

Despite an extended literature about the optimization of eDNA sample analysis to improve detection performance, less attention has been paid to how eDNA sampling design can be optimized. Consequently, there is a wide range of variation in the volume of sampled water among studies, ranging from a few centiliters to tens of liters^14^. Nonetheless, sampling effort is a fundamental aspect for any ecological study or monitoring procedure^17^ and might deeply affect results and interpretations. Some eDNA studies suggested that increasing the volume of sampled water improved the quality of the biodiversity assessment. For example, detection rates of anurans in tropical streams were higher when increasing sampling effort from 20 to 60 liters of water^6^. Moreover, Mächler *et al*.^14^ found a significant positive relationship between the sampled water volume and the detection rate for a macro-invertebrate species. In spite of this, due to financial and technical limitations, a threshold must be fixed in order to optimize eDNA inventories. This consists in determining the best compromise between sampling effort (and its associated financial and time costs) and the accuracy of the biodiversity estimate.

Recently, the sampling effort needed to accurately estimate the fish species richness in temperate lakes has been assessed using spatial replicates and revealed that 5 to 20 liters of water were needed to detect the entire fish fauna^5,8^. However, to date, the optimization of the eDNA sampling effort for the assessment of whole community diversity in running waters (streams and rivers) has never been assessed. A better understanding of this effect will allow optimizing sampling efforts without reducing the quality of diversity estimates. For instance, Nascimento *et al*.^18^ found that the volume of sampled sediments strongly impacted diversity assessments of benthic eukaryotic communities. The stakes of this understanding will be higher in tropical ecosystems, where large sampling efforts are often needed^19^. Indeed, describing tropical communities can be challenging given the wide range of species diversity they host^20^, and the strong contribution of rare species to tropical biodiversity and ecosystem functioning^21,22^.

The aim of this study was to determine the optimal sampling effort for fish inventories using eDNA metabarcoding in tropical streams and rivers. We built on preliminary tests in French Guiana in which 39 freshwater fish communities were sampled using the protocol designed by Valentini *et al*.^10^ for temperate rivers. Those tests showed that one water sample of *ca*. 50 liters permitted to detect a substantial part of the fauna without erroneous detections^23^ (*i.e*. species not expected to occur in the sampled sites according to their known habitat preferences and watershed occurrence). Nevertheless, the standard protocol designed by Valentini *et al*.^10^, did not permit to detect the whole fish fauna of the studied sites^23^ comparing with traditional methods. We hence hypothesized that increasing sampling effort will enhance detection rate. To test this, we filtered water in four highly diverse Guianese streams and rivers using the VigiDNA 0.45 μm; SPYGEN filtering system. In each site we took 10 replicates. Each replicate was collected by filtering for 30 minutes, corresponding to 34 liters of filtered water (standard protocol). We then analyzed how sampling effort (from 34 to 340 liters) affects the estimation of fish biodiversity. Specifically, we sought to define the optimal sampling effort to describe communities through three diversity descriptors: species richness, dissimilarity of species composition and community structure patterns between sites. In addition, two sites were sampled for half of the time (relaxed protocol) than the other four sites to test whether reducing the filtering volume to 17 liters per replicate will degrade the diversity estimates (due to a lower filtered volume), or will improve the results as increasing filtering time can increase the accumulation of PCR inhibitors in the filter^24^.

## Results

### Total biodiversity detected

**Figure 1:**
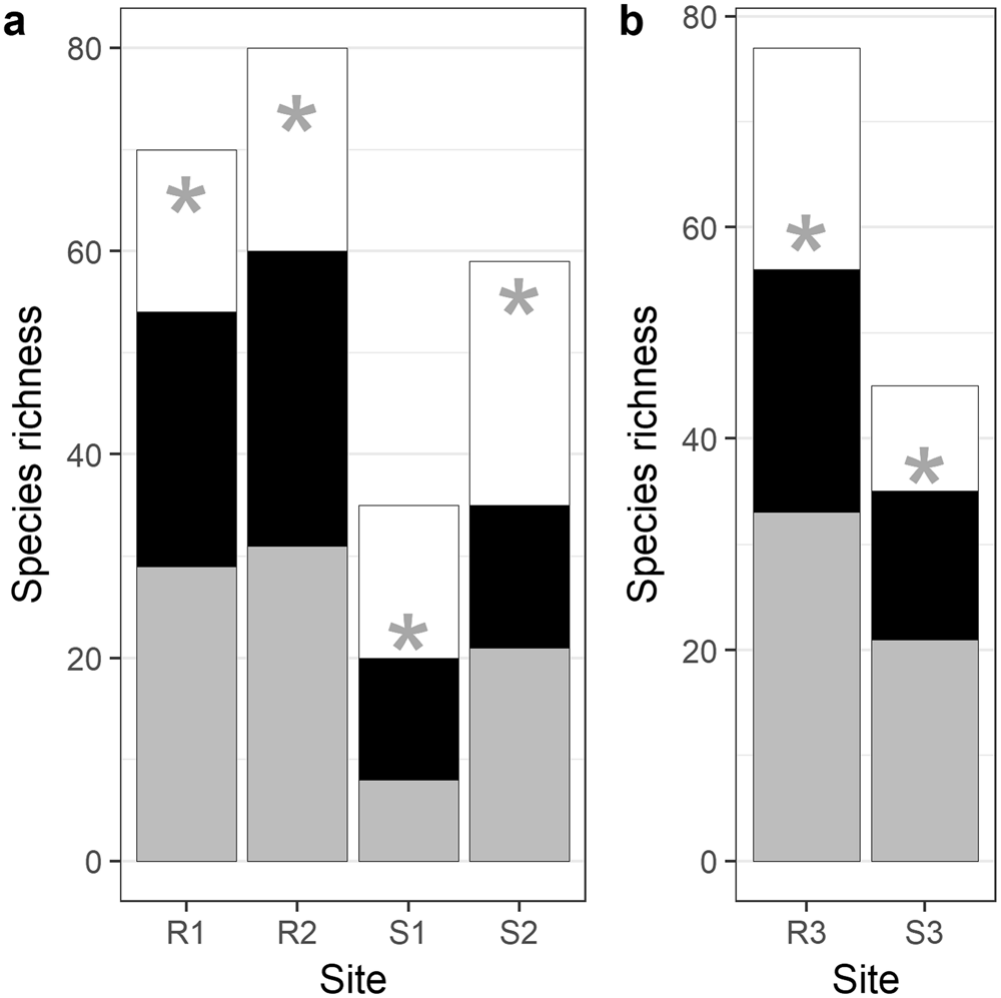
Species richness per site detected with traditional capture-based and eDNA metabarcoding methods with the standard (**a**) and relaxed (**b**) protocols. The species caught only with traditional methods are indicated in white, those detected only with eDNA are indicated in grey, and those detected by both eDNA and traditional methods are indicated in black. The Chao II estimation of species richness using eDNA samples is indicated with a grey asterisk. R1, R2 and R3 are river sites and S1, S2 and S3 are stream sites.

In total, 40,838,558 reads were obtained. After the bioinformatic filtering (see Materials and Methods), 22,488,969 reads were retained, corresponding to 55.1% of the total reads. We found reads in all of the 720 PCR replicates while no reads were found in the extraction and PCR controls. Among all the sites and replicates, we obtained 106 species-level detections, seven genus-level detections, (*Bryconops*, *Guianacara, Krobia*, *Laimosemion*, *Leporinus*, *Moenkhausia*, *Pimelodella*) and two family-level detections(Characidae, Hypopomidae). A total of 279 species occurrences were detected in the six sites. Among those occurrences, only 5 (1.8%) were not consistent with the known distribution of the species per watershed and habitat preference. The total number of detected species per site, when summing across the 10 replicates, ranged from 21 to 60, which accounts for 57 to 83% (on average 71%) of the local fauna derived from fish surveys using both capture-based and eDNA samples (see Materials and Methods) in each site (Fig. 1). A proportion of the undetected species using eDNA are not informed in the molecular reference database (on average 19% of the fauna), but some species were not detected although referenced in our reference database (on average 10% of the fauna). This explains why the Chao II diversity estimator estimated a lower species richness than the combined eDNA and capture-based inventories. Nevertheless, Chao II estimations of species richness using eDNA samples remained consistent with the combined eDNA and capture-based inventories (Fig. 1).

### Replication effects on detected species richness

**Figure 2:**
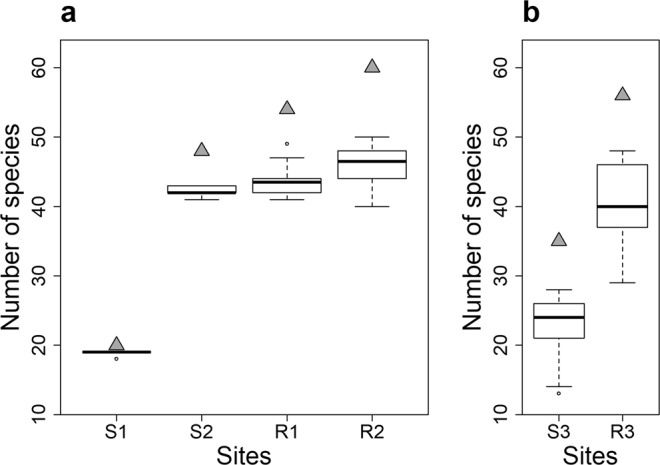
Number of detected species among the ten replicates for each site. Boxplots indicate the number of detected species per replicate. Triangles indicate the total number of species detected in each site (combining the 10 replicates). (**a**) Sites sampled under the standard protocol. (**b**) Sites sampled under the relaxed protocol. R1, R2 and R3 are river sites and S1, S2 and S3 are stream sites.

Under the standard protocol, replicates provided consistent numbers of detected species, as shown by the narrow interquartile ranges in Fig. 2a. This repeatability was particularly marked in stream sites where species richness differed by fewer than three species between replicates. For river sites, species richness varied by up to 10 species between replicates. The number of species found in the sites sampled under the relaxed protocol was less consistent among replicates, with a variation of up to 15 species between replicates for the stream site and up to 19 species between replicates for the river site (Fig. 2b).

**Figure 3:**
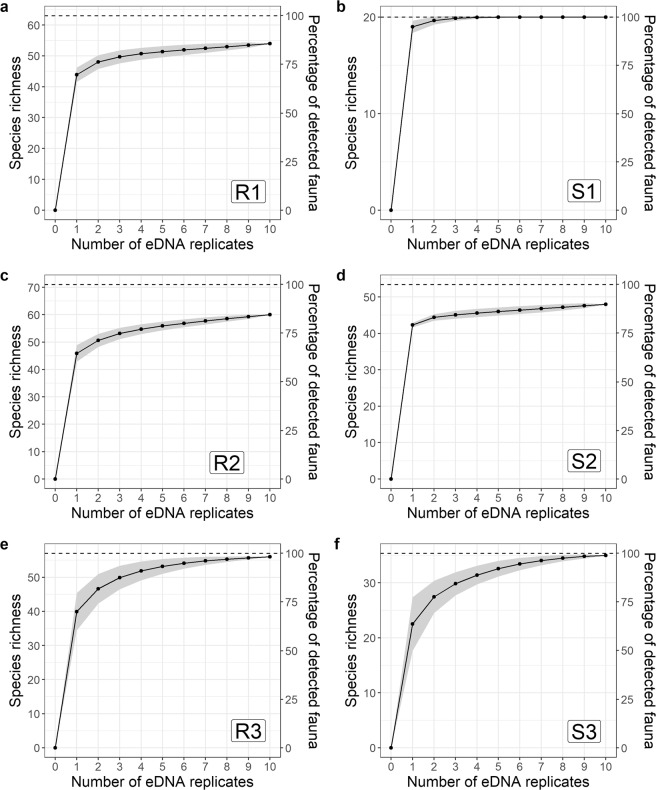
Species accumulation curves (solid lines) with increasing number of replicates for sites sampled under the standard protocol (**a**–**d**) and the relaxed protocol (**e**,**f**). River sites are on the left and stream sites on the right. Confidence intervals are represented by the shaded area. Estimated species richness with the Chao estimator are indicated with a dashed line. The percentage of detected fauna per replicate according to the Chao estimator is represented on the right axis.

With one replicate, detection rate represented 64–95% of the Chao II estimation of expected species richness (Fig. 3). Using the standard protocol, a single replicate detected, on average, 67% of the expected richness in rivers and 87% of the expected richness in streams. Using the relaxed protocol, detection rate was lower in the stream site (*i.e*. 79%), but remained similar to that obtained with the standard protocol in the river site (*i.e*. 69%). Adding a second replicate slightly increased detection rate in sites sampled under the standard protocol, with a gain of less than 4% and 7% in species richness for stream and river sites, respectively (Fig. 3a–d). In contrast, under the relaxed protocol, adding a second replicate increased detection rate by more than 10% (Fig. 3e,f). Finally, increasing sampling effort from three to 10 replicates marginally affected the estimates of species richness using the standard protocol, whereas a substantial gain of species was still observed when increasing the sampling effort with the relaxed protocol. In the latter case, species accumulation curves did not saturate from one to 10 replicates (Fig. 3e,f), while a species saturation was obtained until the second replicate using the standard protocol (Fig. 3a–d). In addition, confidence intervals of the estimated species richness with the relaxed protocol were larger than those obtained using the standard protocol. This indicates that the standard protocol consistently detected similar species richness in the 10 replicates whereas substantial variations among replicates were observed using the relaxed protocol.

### Species composition among replicates

**Figure 4:**
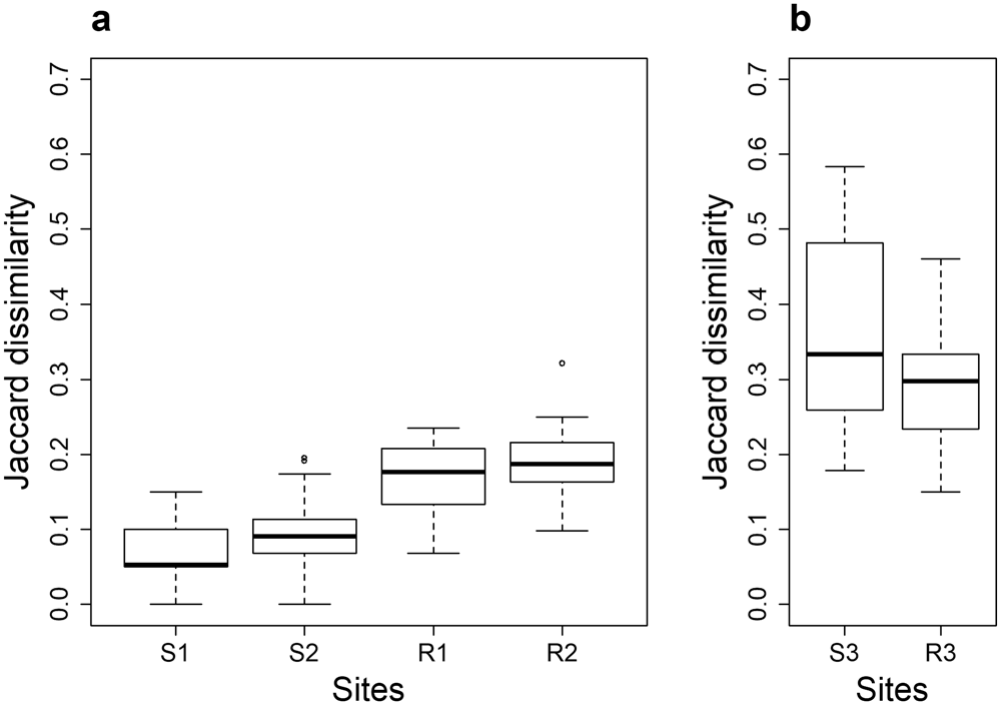
Pairwise Jaccard’s distances between replicates for each site. Boxplots summarize species dissimilarity values (n = 40 per site) among replicates. (**a**) Sites sampled under the standard protocol. (**b**) Sites sampled under the relaxed protocol.

The differences in species composition between replicates were low for the sites sampled under the standard protocol (Fig. 4a). Pairwise Jaccard’s dissimilarity indices ranged from 0.07 to 0.32 for rivers (mean = 0.22) and from 0 to 0.19 (mean = 0.17) for streams, with significantly higher dissimilarity values between replicates in rivers than in streams (Kruskal-Wallis rank sum test: χ² = 27.2; p < 2.2e-16). On average, river faunas differed by 22% between replicates, whereas stream faunas differed by less than 17%. These results contrasted with those obtained using the relaxed protocol (Fig. 4b), which showed a mean species dissimilarity between replicates higher than 30% for both stream and river sites. Accordingly, species dissimilarity between replicates was significantly higher with the relaxed protocol than with the standard protocol (Kruskal-Wallis rank sum test: χ² = 149.76; p < 1.8e-07).

**Figure 5:**
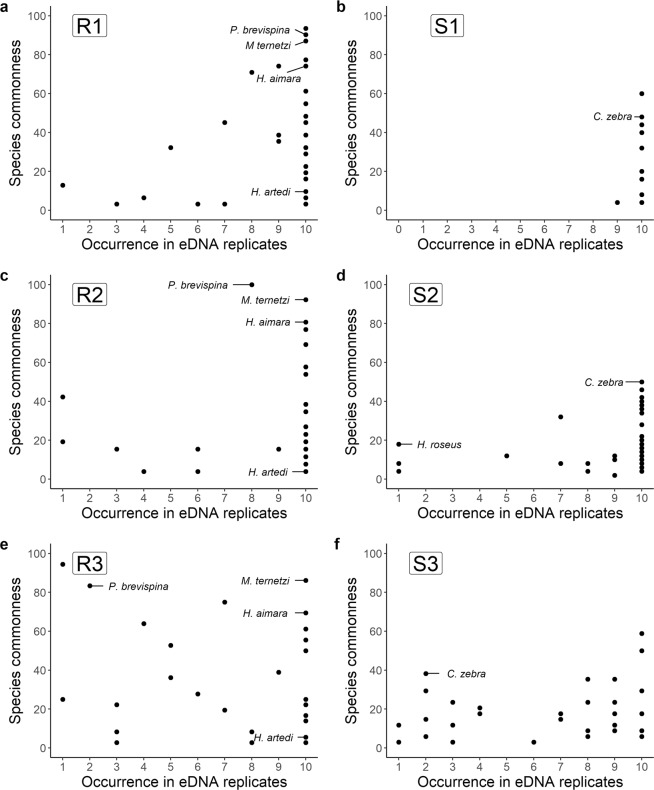
Relationships between the species occurrence in eDNA replicates and the species rarity. Species rarity was measured as the percentage of the occurrence of each species in all the capture-based samples ran in the stream (for stream eDNA sites) or river (for river eDNA sites) stretches of the considered watershed (see methods for details). Some species of interest are indicated on the figure. (**a**–**d**) Sites sampled under the standard protocol. (**e**,**f**) Sites sampled under the relaxed protocol. River sites are on the left and stream sites are on the right.

The species frequency of detection among the eDNA replicates was not influenced by the species commonness in any site. Indeed, common and rare species were systematically detected in all the replicates (Fig. 5). Nevertheless, in the sites sampled under the standard protocol, most of the species that were detected in few eDNA replicates were rare species, given that they were captured in less than 50% of the traditional sampling campaigns. In contrast, using the relaxed protocol, some common species (occurring in more than 60% of the capture-based sampling campaigns) were only detected in a few eDNA replicates.

### Distinguishing assemblages

**Figure 6:**
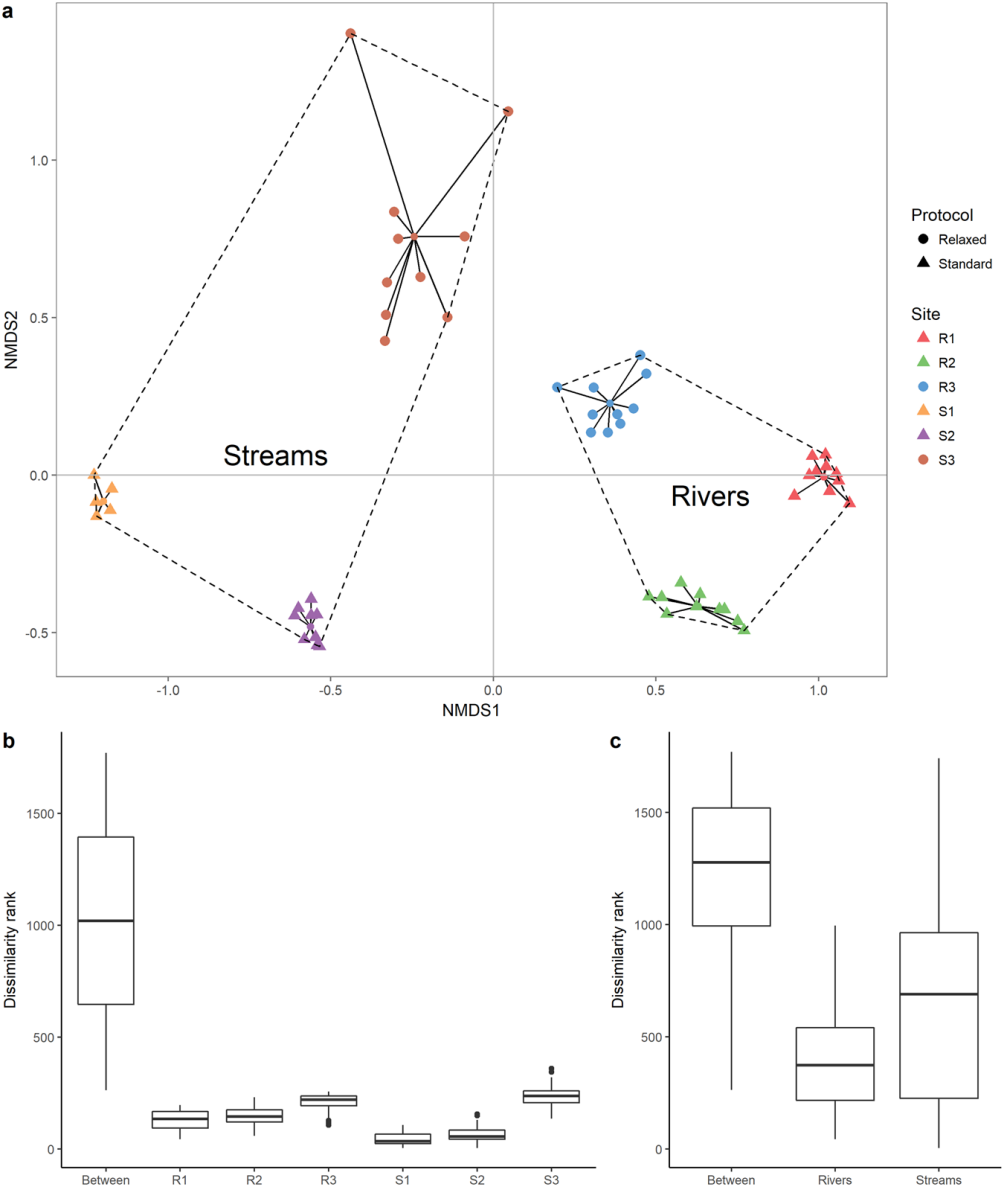
Species composition patterns of the six sites. (**a**) First two axes of the NMDS ordination of the water samples filtered with the standard (triangles) and the relaxed (circles) protocols. The stress of the plot is 0.09. Black segments represents the distance between each replicate and the centroid of their respective site in the two-dimensional space. The dashed lines indicate convex hulls grouping stream and river sites. (**b**) Boxplots indicate the dissimilarity ranks values between and within sites. (**c**) Boxplots indicate the dissimilarity ranks values between and within habitats.

The first two axes of the NMDS provided a good two dimensional representation of the replicates according to their species composition (Fig. 6a), as the stress of the plot was lower than 0.1^25^. The ordination distinguished sites, without overlap between replicates from different sites (ANOSIM statistic R = 0.996; p < 0.001, Fig. 6b), in spite of a more pronounced dispersion of the replicates collected under the relaxed protocol. Furthermore, the fish composition of the river sites were significantly distinct from those of the stream sites (ANOSIM R = 0.996, p < 0.001, Fig. 6c), as shown by the separation of the stream and river sites throughout the first axis of the NMDS (Fig. 6a).

## Discussion

The eDNA metabarcoding approach has been claimed as an efficient tool to obtain inventories of aquatic organisms^10^, but the optimal sampling effort to get those inventories has never been investigated in running waters. Here we show that eDNA replicates not only have a high repeatability on the estimation of species richness but also on the identity of the detected species, which both exhibited slight variations among replicates. Besides, the fish fauna detected in each site was consistent with the one known from each river basin^26–28^ giving that the fish fauna of French Guiana is spatially structured into several freshwater ecoregions^29^. Moreover, our results are also consistent with the habitat preferences (streams vs rivers) of Guianese fishes^26–28^. The rare erroneous detections (1.8% of the detections) were already reported as the result of an incompleteness in our molecular reference database^23^. Indeed, a few species not included in the molecular reference database were erroneously assigned to their closest relative available in the reference database. Furthermore, the fish fauna derived from the eDNA method accounted on average for 71% of the inventoried fauna from each site whereas capture-based methods detected on average 61% of the inventoried fauna, making eDNA more efficient than traditional capture based methods. Nevertheless, discrepancies remain between methods, and none can provide an exhaustive image of the local fauna due to the technical limitations of the sampling methods. For instance, capture-based methods are size and species selective^30^, whereas eDNA detection ability is limited by the completeness of the reference database. Therefore, capture-based and eDNA methods complement each other and should be combined to get the most realistic image of the fauna.

The standard protocol, consisting in the filtration of 34 liters of water, provided little variation in the species richness and in the species composition among replicates. Those trends were more marked in stream sites where replicates gave consistent number and identity of the detected species, with no more than two species differing among replicates. Conversely, in rivers, the differences between replicates reach up to 10 species, suggesting that the sampling effort needed to survey all the detectable species may be less important in streams than in rivers. Certainly, higher species richness is expected in rivers than in streams, given that larger areas are expected to offer more niches and habitat space and potentially host more species and larger population sizes^31^. Indeed, this trend was confirmed in freshwater ecosystems, where species richness increases from upstream to downstream^32,33^. Accordingly, the volume of water needed to get a realistic image of the fauna should increase with the size of the system.

For both stream and river sites, a substantial part of the fish fauna was recovered with only few eDNA replicates using the standard protocol. On average, 87% of the expected fauna from small streams, counting 21 to 48 species, was detected with a single replicate of 34 liters. Adding a second replicate (*i.e*. 68 liters of water) enhanced this detection up to 91%. For river sites, a single replicate was sufficient to detect 67% of the fauna, counting 54 to 60 species, and adding a second replicate enhanced the detection rate up to 74%. In addition, in the four sites most of the species were systematically detected in 100% of the eDNA replicates. This part of the fauna detected in all replicates included both common and rare species. For instance, *Hoplias aimara* or *Myloplus ternetzi*, two common and widespread fish species in French Guiana rivers^27^, were detected in all the eDNA replicates of all the river sites. Similarly, *Hypopomus artedi* and *Sternopygus macrurus*, although rarely captured in rivers, are known to have colonized all the major watersheds of French Guiana^27^ and were consistently detected in all of the eDNA replicates of the rivers. In addition, the few species not systematically detected in all the eDNA replicates of a given site were rare species, such as *Hyphessobrycon roseus* in site S2, an uncommon species in French Guiana^27,28^. This parallels Mächler *et al*.^14^ results, showing that the detection of a rare macro-invertebrate species needs a higher sampling effort than the detection of the common species. Likewise, Lopes *et al*.^6^ showed that increasing sampling effort resulted in an increase of 41% of the detection rates for rare species and of only 8–15% for common species of amphibians in tropical rivers. Consequently, although a trend towards species saturation after 68 litters of water, if the purpose is to exhaustively inventory the fauna, it will be required to filter more than 68 liters to improve the detection of rare species.

Our study offers guidelines to optimize and standardize the volume of filtered water in eDNA studies without reducing the representativeness of the fauna. Previous studies in temperate and less diversified ecosystems, showed a strong heterogeneity in the sampling effort needed to obtain an exhaustive image of the fish fauna. For example, 16 liters of water were sufficient to detect 16 of the 18 historically recorded species in a temperate stream^9^. Similarly, Evans *et al*.^5^ estimated that 5 liters of water are needed to accurately estimate the fish species richness in a small freshwater reservoir colonized by 21 fish species, and Hänfling *et al*.^8^ considered that filtering 20 liters of water was sufficient to identify 14 of the 16 species inhabiting an English lake. Conversely, Civade *et al*.^7^ and Valentini *et al*.^10^ filtered very large water volumes (up to 6 samples of 34 liters and 6 samples of 60 liters per site, respectively) to detect nearby 20 species in European rivers. We illustrated that filtering intermediates water volumes (2 samples of 34 liters), is sufficient to get a representative picture of the fish fauna inhabiting our sites. Consequently, we recommend using two replicates of approximately 34 liters to sample species rich communities in tropical running waters.

We advise not to reduce the filtering volume per replicate below 34 liters, since reducing filtering volume by 50% (filtering 17 liters instead of 34 liters during 15 minutes instead of 30 minutes) increased the discrepancy between replicates in terms of both species richness and species identity. Moreover, sampling a lower water volume per replicate (relaxed protocol) resulted in replicates missing common species. For instance, *Characidium zebra*, frequently found in Guianese streams^32^ or *Poptella brevispina*, occurring in almost all the capture-based samples from the rivers, were not systematically detected with the relaxed protocol (sites S3 and R3, respectively), whereas they were frequently detected in the sites sampled under standard protocol. Therefore, our results underline the need to collect a sufficient volume of water to get reliable and repeatable estimates of fish diversity. It might also be proposed to replace the two replicates by a single filtration of more than 34 liters to reduce the financial costs, but this might be risky due to filter clogging by suspended material. Our trials to increase filtered water volume per replicate, led to damage either the filter, the peristaltic tube or the peristaltic pump. We thus discourage increasing filtered water volume per replicate over 34 liters, with the used materials.

Although 68 liters of water were needed to detect most of the fauna, a single replicate of 34 liters was enough to identify the core of fish assemblages and therefore distinguish between sites and between ecosystem types (stream *versus* rivers). In spite of the close proximity of the sites sampled under the relaxed protocol (only separated by nearby 300 meters), the eDNA data distinguished R3 and S3 sites. Notably, we did not observed any trend toward nestedness of the stream fauna within the riverine fauna. This indicates that even though streams and rivers have been suggested to act as conveyor belts of eDNA^34^, the DNA flowing through the water might not be conserved between distant sites. Therefore, our results reinforce the idea of a detection distance of the eDNA limited to 500 meters in flowing waters, as shown by Jane *et al*.^35^. Forthcoming studies should specify to which extent distance detection of eDNA in the water and species detection rate vary between tropical and temperate ecosystems. Indeed, physiochemical factors such as temperature, pH, conductivity or UV radiation can impact DNA degradation and transport^15,16^. Nevertheless, our results highlight the ability of eDNA to inventory local species assemblages in tropical running waters, limited up to now to temperate environments^7,36,37^.

The eDNA approach using the standard sampling protocol deserves to be applied to ecological and conservation studies of highly diverse ecosystems such as tropical waters. Its applicability to Guianese freshwater ecosystems is of particular interest since current fish sampling methods vary among ecosystems, besides being time consuming, destructive and species selective. Indeed, both rotenone sampling in streams and gillnet sampling in rivers are destructive for fishes^38^, and collect fish from a limited range of habitat resulting in partial images of the fauna^23^. In opposition, eDNA sampling was efficient in both streams and large rivers thereby standardizing the potential sampling bias among ecosystems and making possible to compare stream and river samples. Going further in the development of the eDNA inventories requires to complement the reference database to consider more species and to avoid rare, but still existing, false detections. Another forthcoming issue, might be to improve the distinction between closely related species using multiple molecular markers, and by optimizing bioinformatics protocols as proposed by Hänfling *et al*.^8^ and Evans *et al*.^5^. Moreover, DNA releases may vary among species and affect detection rate, and it would therefore be useful to test for phylogenetic, functional and behavioral signals in species detectability. Finally, as stated before, a plethora of protocols has emerged for every step of the eDNA procedure. For the collection of DNA from water samples, three common protocols are used: filtration^10^, precipitation^39^ and centrifugation^40^. The filtration method, consisting on filtering large volumes of water, has proved to yield higher detection rates compared to other methods in both natural ecosystems^12^ and laboratory conditions^13^. Here we tested one specific filtering system VigiDNA 0.45 μm; SPYGEN, le Bourget du Lac, France, but alternative filtering systems may require different sampling efforts due to differences in filter types and pore sizes^41,42^. Therefore, the optimal water volume to obtain robust diversity estimates may vary with the used system and collection method. This highlight the need of forthcoming studies comparing the performance of different filtering systems to gain a more comprehensive view on the performance of the eDNA metabarcoding method in aquatic environments.

## Materials and Methods

### eDNA sampling

This study was conducted in French Guiana in November 2016 (during the dry season). This territory is subjected to an equatorial climate, and is covered by a dense primary rainforest. Freshwater bodies in this country host nearby 405 fish species^26^, making Guianese freshwater ecosystems and excellent place to optimize eDNA sampling effort in species-rich communities. Six sites corresponding to three small streams and three rivers, were sampled. Stream sites (S1, S2, and S3) are less than 10 meters wide and 1 meter depth whereas river sites (R1, R2, and R3) are wider than 30 meters and deeper than 1 meter. Those sites belong to distinct watersheds (Mana (S1); Maroni (S2); Comté (R1); Sinnamary (R2); Approuague (S3, R3)). They are free from human settlements upstream and are therefore little affected by human activities (See Supplementary Table S1 for more details on localities and their characteristics).

At each site, 10 filtrations were realized in the same place, resulting in 10 field replicates per site. Each filtration was done following Valentini *et al*.’s^10^ protocol for running waters. Per replicate, we filtered 34 liters of water during 30 minutes in four sites (S1, S2, R1 and R2). In two complementary sites (S3 and R3) we filtered 17 liters of water during 15 minutes. This resulted in two different treatments called “standard protocol” and “relaxed protocol” respectively. This permitted to test if filtering volume can be optimized without decreasing detection performance. For each replicate, a peristaltic pump (Vampire sampler, Burlke, Germany) and a single-use tubing were used to pump the water into a single-use filtration capsule (VigiDNA 0.45 μm; SPYGEN, le Bourget du Lac, France). The input part of the tubing was placed few centimeters below the surface in zones with high water flow as recommended by Cilleros *et al*.^23^. Sampling was achieved in turbulent area (rapid hydromorphologic unit) to ensure an optimal homogenization of the DNA throughout the water column. To avoid DNA contamination among sites, the operator always remained downstream from the filtration area and stayed on the bank (for streams) or on emerging rocks (for rivers). At the end of the filtration, the filtration capsule was emptied of water, filled with 80 mL of CL1 conservation buffer (SPYGEN) and stored in individual sterile plastic bags kept in the dark. Samples were then stored at room temperature for less than one month before DNA extraction.

### Laboratory and bioinformatics analyses of eDNA

For DNA extraction, each filtration capsule was agitated for 15 min on an S50 shaker (cat Ingenieurbüro™) at 800 rpm and then emptied into a 50-mL tube before being centrifuged for 15 min at 15,000 × g. The supernatant was removed with a sterile pipette, leaving 15 mL of liquid at the bottom of the tube. Subsequently, 33 mL of ethanol and 1.5 mL of 3 M sodium acetate were added to each 50-mL tube and stored for at least one night at −20 °C. The tubes were centrifuged at 15 000 × g for 15 min at 6 °C, and the supernatants were discarded. After this step, 720 µL of ATL buffer from the DNeasy Blood & Tissue Extraction Kit (Qiagen) was added. The tubes were then vortexed, and the supernatants were transferred to 2-mL tubes containing 20 µL of Proteinase K. The tubes were finally incubated at 56 °C for two hours. Afterwards, DNA extraction was performed using NucleoSpin® Soil (MACHEREY-NAGEL GmbH & Co., Düren Germany) starting from step six and following the manufacturer’s instructions. The elution was performed by adding 100 µL of SE buffer twice. Four negative extraction controls were also performed. They were amplified and sequenced in the same way as and in parallel to the field replicates to monitor possible laboratory contaminants. After the DNA extraction, the samples were tested for inhibition by qPCR following the protocol in Biggs *et al*.^43^. If the sample was considered inhibited, it was diluted 5-fold before the amplification.

We performed DNA amplifications in a final volume of 25 μL including 1 U of AmpliTaq Gold DNA Polymerase (Applied Biosystems, Foster City, CA), 10 mM of Tris-HCl, 50 mM of KCl, 2.5 mM of MgCl2, 0.2 mM of each dNTP, 0.2 μM of “teleo” primers^10^ and 3 μL of DNA template. We also added human blocking primer for the “teleo” primers with a final concentration of 4 μM and 0.2 μg/μL of bovine serum albumin (BSA, Roche Diagnostic, Basel, Switzerland) to the mixture. We realized 12 PCR replicates per field replicate. The forward and reverse primer tags were identical within each PCR replicate. The PCR mixture was denatured at 95 °C for 10 min, followed by 50 cycles of 30 s at 95 °C, 30 s at 55 °C and 1 min at 72 °C and a final elongation step at 72 °C for 7 min. This step was done in a room dedicated to amplified DNA with negative air pressure and physical separation from the DNA extraction rooms (with positive air pressure). We also amplified the four negative extraction controls and three PCR negatives controls (with 12 replicates as well) and sequenced them in parallel with the 720 PCR replicates (6 sites, 10 field replicates per site and 12 PCR replicates per field replicate). We pooled the purified PCR products in equal volumes to achieve an expected sequencing depth of 500,000 reads per sample before the libraries preparation. Five libraries were prepared using the Metafast protocol (https://www.fasteris.com/metafast), a PCR-free library preparation, at Fasteris facilities (Geneva, Switzerland). Sequencing were performed using an Illumina HiSeq2500 (2 × 125 bp) (Illumina, San Diego, CA, USA) and the HiSeq SBS Kit v4 (Illumina, San Diego, CA, USA) following the manufacturer’s instructions at Fasteris facilities (Geneva, Switzerland).

The sequence reads were analyzed using the programs in the OBITools package (http://metabarcoding.org/obitools)^44^ following the protocol described in Valentini *et al*.^10^. The ecotag program was used for the taxonomic assignment of molecular operational taxonomic units (MOTUs) using a threshold of 98% of identity with the reference database available from Cilleros *et al*.^23^, that counts 130 Guianese fish species. The GenBank nucleotide database was checked but Guianese fishes being poorly informed (most of the sequences are from Cilleros *et al*.^23^), it did not provided additional information in our case. We discarded all MOTUs with a frequency of occurrence below 0.0003 per library in each sample, considered as tag-jumps^45^. These thresholds were empirically determined to clear all reads from the extraction and PCR negative controls included in our global data production procedure as suggested by De Barba *et al*. 2014^46,47^.

### Comparisons with traditional capture-based methods

All the capture-based samplings were achieved during the dry season from 2008 to 2016 as part of research and biodiversity management programs supported by the French ministry of environment (DEAL), the French Guyana National park (PAG), and the French National Center for Scientific Research (CNRS). Stream fishes were sampled using rotenone, following the protocol described by Allard *et al*.^48^. Riverine fishes were sampled using a standardized gill-net protocol designed by Tejerina-Garro and De Mérona^49^. Since neither rotenone nor gill-net samples provide an exhaustive image of the fish fauna^23^, we combined local inventories using gillnets and rotenone available in each site and eDNA results to estimate the overall fauna inhabiting each site.

Additionally, we compared the occurrence of species in the eDNA replicates with the commonness of the species. Since absolute commonness values are not available, the percentage of occurrence of each species in the watershed was used as a surrogate to species commonness (species occurring in more than 50% of the sampling occasions) or rarity (species occurring in less than 50% of sampling occasions)^50^. More specifically, we compared in each site, the percentage of eDNA replicates in which a species was detected against the percentage of sites in which the species was captured though all the capture-based sampling campaigns ran since 2008 in the stream or river stretches of the considered watershed for stream and river eDNA sites, respectively. In streams, the captures were realized in 25, 50, and 34 sites in the Mana, Maroni and Approuague watersheds, to which sites S1, S2 and S3 belong, respectively. In rivers, the captures were realized in 31, 26, and 36 sites in the Comté, Sinnamary and Approuague watershed, to which sites R1, R2 and R3 belong, respectively.

### Statistical analyses

The obtained sequences were used to build a presence/absence matrix per field replicate and per site, in which only taxa detected to the species level were incorporated. Species accumulation curves^17^ with confidence intervals were drawn for each site using the *speccacum* function to examine the impact of replication on the number of species detected. Additionally, expected species richness and confidence intervals were calculated for each site using the Chao II estimator^51^. This allowed to estimate the detection rate (*i.e*. the percentage of detected fauna with the eDNA) according to sampling effort (from one to ten replicates per site). The dissimilarity in species composition among replicates was assessed by calculating pairwise Jaccard’s distances with the *vegdist* function. Then, the dissimilarity values were ordinated using non-metric multidimensional scaling (NMDS) to visualize how replicated eDNA data discriminate sites and habitat (streams vs. rivers) patterns and to determine the sampling effort needed to identify community changes among sites. Differences in species compositions between sites and habitat types were statistically tested by permutational analysis of similarities (ANOSIM). This analysis tool allows to test the statistical significance of dissimilarity between groups comparing to the within groups dissimilarity using the rank of dissimilarity values^25^. All the statistical analyses were performed in R^52^ using the vegan package version 2.4–4^53^.

## Supplementary information

Table S1

## Data Availability

All Illumina raw sequence data and the species presence/absence matrix per field replicate and per site are available.
