# Supplementary material for: Optimizing environmental DNA sampling effort for fish inventories in tropical streams and rivers
Source: Sci Rep. 2019 Feb 28;9:3085. doi: 10.1038/s41598-019-39399-5 (PMC6395586; doi:10.1038/s41598-019-39399-5)
Supplement: Supplementary file 1 — Table S1 [file 41598_2019_39399_MOESM1_ESM.pdf]

Supporting information for

Optimizing environmental DNA sampling effort for fish inventories in tropical streams and rivers

Cantera Isabel[1], Cilleros Kévin[1], Valentini Alice[2], Cerdan Axel[1,5], Dejean Tony[2], Iribar Amaia[1], Taberlet Pierre[3], Vigouroux Régis[4] and Brosse Sébastien[1]

Code	Site name	Watershed	Width (m)	Latitude	Longitude	Distance from the source (km)	Sampling protocol
S1	Crique à l'est	Mana	3.4-3.97	3.6626	-53.222	4.1	Standard
S2	Point chaud	Maroni	4.1-10	3.6081	-53.1741	8.3	Standard
S3	Crique Museum	Approuague	1.7-5.5	4.0391	-52.6769	4.7	Relaxed
R1	Lysis	Comté	45-55	4.5145	-52.5149	89.3	Standard
R2	Saut dalles	Sinnamary	30-40	4.5527	-52.8983	124.6	Standard
R3	Aratai	Approuague	30-40	4.0331	-52.6994	95.5	Relaxed

Table S1: Site chatacteristics: site local name, watershed membership, average width in meters, site position (WGS84) and distance from the source in kilometres. The standard sampling protocol consists in collecting eDNA from 34 litres of filtered water whereas the relaxed protocol consists in collecting eDNA from 17 litres of filtered water.
